# Adaptive Spatial Division-Guided Resource-Based Economic Transformation with Synergistic Resource, Economic, and Environmental Health

**DOI:** 10.1155/2022/6799633

**Published:** 2022-05-31

**Authors:** Jiangong Yang

**Affiliations:** School of Economics Lanzhou University, Lanzhou 730000, China

## Abstract

Relying on the advantages of its input factors, the resource-based economy has achieved rapid development. However, with the global emphasis on scientific development, various contradictions have sharply reduced the competitiveness of the resource-based economy. In the process of a new round of changes in the world economic pattern and the adjustment of China's development strategy, most resource-based economies have begun to implement transformation actively or passively. Resource-based economy was mostly established and developed in the period of planned economy, which made great contributions to national construction and accomplished brilliant achievements in regional economic and social development. However, the development of natural resources always goes through stages of development, growth, maturity, and decline. Therefore, resource-based cities also have problems of rise, prosperity, and decline. From the perspective of adaptive space division, this paper analyzes and studies the teaching of the coordinated development of resource-based economy in the transformation of the resource-based economy and environment economy and puts forward the panel data model, logistic curve, and other algorithm models. After optimization, the collaborative development model is designed. Based on the analysis of the model, it is found that the error analysis of the model has improved by 77.3% and the average growth rate of transformation benefits is generally 56.8%.

## 1. Introduction

As the source of basic energy and important raw materials in my country, resource-based economy has made outstanding contributions to the development of the national economy [1]. The question of how to avoid repeating the mistakes; develop high-efficiency industries based on resource advantages; scientifically demonstrate, optimize, and adjust economic development from the source of decision making; and protect economic ecology and environment while developing the economy has become a key problem that needs to be solved in the development of the emerging resource-based economy [2]. Resource-based economy is mostly established in the desolate backcountry, which is mostly old, small, and poor areas [3]. My country is a rare resource-rich country in the world, with a wide variety of resources, among which coal, iron, oil, and other resources are exploited on a relatively large scale, which plays an important role in the formation and development of their corresponding resource-based economy [4]. Since the establishment of New China, due to the large-scale demand for energy and raw materials in national economic construction, the construction of the resource development base has received great attention [5]. The rise and development of the resource-based economy has provided a large number of employment opportunities for these areas and accelerated the process of economization [6]. The high concentration and large-scale development of resource-based industries have greatly changed the economic development mode and lifestyle of their surrounding areas and promoted the development of regional economy [7].

The transformation of resource-based economy is a worldwide issue. This type of economy rises or develops due to the exploitation of natural resources and also stagnates or even declines due to the reduction and depletion of natural resources [8]. The operability of sustainable development is based on regional sustainability. Resource-based economy plays an important role in China's national economic and social development [9]. The nonrenewable nature of natural resources makes the development of resource-based economy face more difficulties than other economies [10]. My country is now in an important period of economic development and transformation. It is necessary to guide the direction of economic development with the concept of green and ecological projects and to build an ecologically civilized society as the goal of economic development. Only when the economic development model is successfully transformed can we build an ecological civilization. This strategic goal has become a reality [11]. In general, the environmental scheduling problem is to optimize the economic minimization of resource scheduling as a single objective by analyzing and using the constraint model of power scheduling. However, these methods cannot effectively solve the nonconvex Pareto optimal problem. At present, the multiobjective optimization algorithm that simultaneously processes two or more objectives in parallel has been applied to the multiobjective environmental and economic scheduling problem [12]. The core of building a conservation-minded society is to conserve resources, that is, in all aspects of production, circulation, consumption, and other fields, through the adoption of comprehensive measures such as technology and management, strict conservation, continuous improvement of resource utilization efficiency, and reduction of resource consumption and environmental costs as much as possible. A development model that meets people's growing material and cultural needs was needed [13]. Environment-friendly society is a social form of harmonious coexistence between man and nature. The relevant research mainly adopts the research means of economic geography, and the application of management theory and economic analysis methods is very few. The research method is relatively single, so it is urgent to strengthen the normative theoretical research [14]. In addition, the resource-based economy relies too much on the resource-based industries and forms a tie relationship with the resource-based industries.

This paper will examine the special problems and special laws in the sustainable development of resource-based economy, as well as the theory and method of sustainable development of the resource-based economy and the main methods of implementation, based on the basic theory of sustainable development and the actual development of resource-based economy. From the standpoint of sustainable development, it will present theoretical foundations and operational decision-making suggestions for my country's resource-based economy [15]. However, in the transformation of resource-based economy based on adaptive space division, the previous research did not perform a good optimization analysis on the coordinated development of resource-based economy and environment. However, in the transformation of the resource-based economy, it is critical to solve or improve the problem or process of coordinated development of resource-based economy, environment, and economy. As a result, the following innovations are proposed in this paper:

1. This paper puts forward a new mode of industrial development of resource-based economy: intensive-green-chain network development mode. Based on the analysis of the coordinated development of resources, environment, and economy in resource-based economy transformation, this paper studies the connotation and characteristics of transformation based on the theory of sustainable development, analyzes the feasibility of transformation and the theoretical conditions of sustainable development, and designs a dynamic development model on this basis.
2. This paper makes a multiobjective analysis on the coordinated development of resources, environment, and economy in the resource-based economy transformation in the adaptive space division because the adaptive space division solves the problem of different subspaces in the multiobjective optimization problem. In the experimental analysis, it can be divided into a large number of subspaces, and several important theories such as the panel data model algorithm are proposed in the algorithm to obtain the comparison of these algorithms in detection.

## 2. Related Work

In order to improve the level of intensive and friendly use of the construction land in the Chang Zhu Tan area and realize the sustainable use of land resources, Feng et al. [16] first analyzed the relationship between “two oriented” social construction and intensive and friendly use of construction land, explained the connotation of saving and friendly use of construction land, and then constructed the evaluation index system of intensive and friendly use of construction land. The factor analysis method is used to dynamically analyze the evolution trend of intensive and friendly utilization of construction land in the Chang Zhu Tan area [16]. Stevenson et al. [17] proposed a multiobjective particle swarm optimization algorithm based on bi-local optimization and combined it with the constraint processing method with the best feasible solution to solve the multiobjective EED problem [17]. Halder et al. [18] and others believe that efforts should be made to form a scientific form of economic development mode, social development mode, rational economic structure, and spatial pattern of resources and environment that do not waste resources and rather improve environmental quality. On the basis, the waste of resources and environmental damage are completely reduced [18]. The study by Morais et al. [19] shows that improving the quality of social development is the objective requirement of social development under the background of the new normal. Only by improving the quality of economic development can we promote the smooth transformation and optimization of social and economic structure, the improvement of development quality, and the improvement of ecological civilization construction performance [19]. Song et al. [20] and others think that after entering the post-industrialization, the status of natural resources as the main factor of production has been shaken, and the dependence of resource-based economy on resources and the constraints of resources on economy have become the bottlenecks of this economic growth. Due to the restriction of the life cycle of natural resources development and the gradual shrinking of the traditional product market, the resource-based economy began to decline, even faced with extinction, and the resource-based economy gradually changed from “core economy” to “marginal economy” [20]. Wang et al. [21] proposed that in multitarget tracking systems, the number of targets is usually unknown and constantly changing, and each target is in constant motion. The MTT algorithm plays a vital role in the multitarget tracking system. Through the target information obtained from sensors, such as position information, target attributes, and target strength, it can achieve stable positioning and tracking of multiple targets [21]. The research results of Chen and Guo [22] show that if economic growth continues at the expense of environment and quality, it will be difficult for resources and environment to bear its weight. After experiencing the rapid growth and re-industrialization since this century, China's economy has also reached an important juncture of transformation and upgrading [22]. Yu et al. [23] and others think that “the practical goal of building socialist ecological civilization is to build a sustainable modern economic model that is coordinated between ecology and economy, which is mainly manifested in developing circular economy, building an eco-safe economy and society, and realizing the development of ecological green civilization” [23]. The research of Chatterjee and Dutta [24] explained that the economy is an inevitable product of the development of human society and economy. The process of industrialization and economization in our country is accelerating, and a large number of rural surplus labor are transferred to the economy, which makes the economy quickly gather a large number of people. The rapid expansion of the economic scale and the rapid expansion of the population have brought problems such as water and soil resources' shortage and ecological damage [24]. Babamiri and Marofi [25] analyzed the country as a whole. The results show that the problems of forest land loss and sustainable forest management are related to the number of state-owned public stops and the differences between developed and developing countries. This gap is also an important factor affecting the sustainable development of state-owned forests [25]. Vondolia et al. [26] think that under the multitarget system, due to the problems of missed detection, clutter measurement, the emergence and extinction of targets, the simultaneous matching of motion models for multiple targets, and the unknown correspondence between multiple targets and measurements, all these will have a great impact on the performance of multitarget estimation [26]. The research of Pang et al. [27] and others shows that the leading industries of resource-based economy are developed on the basis of local advantageous resources. This resource-oriented development model is in the early stage of industrialization and economization, and the resource market supply has its practical significance under the condition of shortage [27]. Marin G, Marino M, and Pellegrin C believe that the two oriented development of industry refers to taking technological innovation and management innovation as the means and for the purpose of improving economic, social, and ecological environmental benefits, promoting the development of the industrial system in the direction of low resource consumption and less environmental pollution, so as to optimize the industrial structure, enhance the sustainable development capacity of the industry, and make it meet the requirements of the construction of two oriented society; we further constructed the evaluation index system of the development level of two oriented industry and conducted an empirical analysis [28].

On the basis of the above related research, the positive role of adaptive spatial division of labor in the coordinated development of resources, environment, and economy in the transformation of resource-based economy is determined, and a new strategy for coordinated development of resources, environment, and economy is constructed. Based on the spatial adaptive division of the resource-based economy in transition, in-depth analysis and research were conducted on the coordinated development of resources, environment, and economy, with a view to making more effective use of resources, exploring the hidden value behind resource data, and identifying potential problems affecting the coordinated development of resources, environment, and economy in the transformation of a resource-based economy.

## 3. Methodology

### 3.1. Research and Analysis of Related Theories

#### 3.1.1. Adaptive Spatial Partitioning

The adaptive multiobjective evolutionary algorithm based on the division of solution space solves the multiobjective optimization problem. First, the solution space of the multiobjective optimization problem is divided into a large number of subspaces. In the process of algorithm evolution, each subspace retains a nondominated solution set to ensure the diversity of the population. From this, we can know the general continuous multiobjective optimization model:

(1) $$\begin{array}{c} \text{Minimize }F\left(x\right)=\left(f_{1}\left(x\right),f_{2}\left(x\right),\ldots ,f_{m}\left(x\right)\right). \end{array}$$

*x*=(*x*_1_, *x*_2_,…, *x*_*n*_) ∈ *R*^*n*^. represents a decision vector, Ω is the decision space, and *F* : Ω⟶*R*^*m*^ is an objective space composed of *m* objective functions *f*_1_, *f*_2_,…, *f*_*m*_. For adaptive space partitioning, all the original frames are basically closed, and all the objective functions are continuous functions of their decision vectors. The completion time and cost of workflow are two conflicting optimization objectives, which we call multiobjective optimization problems. According to the value characteristics of variables, multiobjective optimization problems can be roughly divided into continuous multiobjective optimization problems and discrete multiobjective optimization problems. The multidimensional search space is divided into multiple grids. The particles in the grid adjust their speed and position by sharing the empirical information of “guiding” particles.

#### 3.1.2. Resource-Based Economy and Resource-Environment-Coordinated Economy

**Figure 1:**
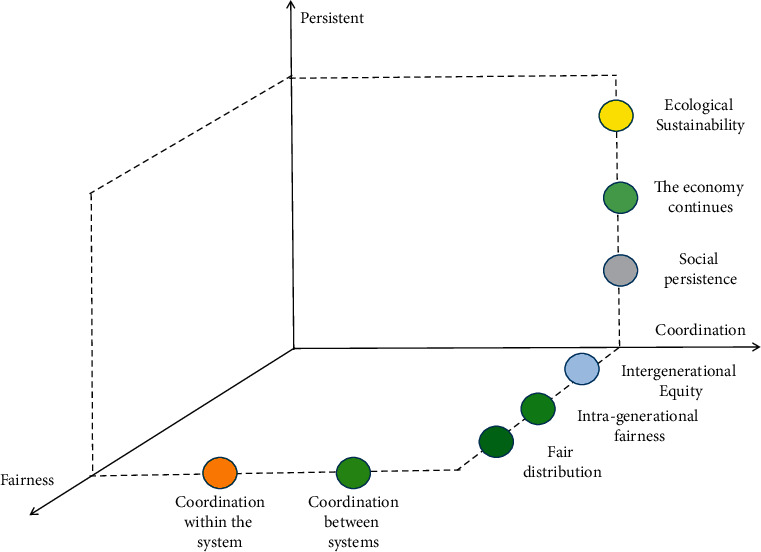
Basic connotation of sustainable development.

Generally speaking, a resource-based economy is a kind of specialized functional economy, which refers to the economy that rises with the development of resources, or the economy that thrives again due to the development of resources in its development process. As a special type of economy, an economy built or developed basically relying on resource development, its leading industries are extractive industries and primary processing industries built around resource development. The industrial development mode of “high consumption, high investment, and high pollution” of the resource-based economy has many disadvantages. While developing the economy, it leads to the excessive consumption of natural resources and the destruction of ecological environment. It is an unsustainable production and consumption mode. The mechanism behind the course of resources is actually very simple, that is, under severe pressure, resource-poor economies have to abandon the traditional growth mode, adopt technological innovation and institutional innovation, and embark on a new economic development route. The sustainable development theory, system theory, and self-organization theory have provided us with good inspiration for understanding and managing economic problems and provided us with basic ideas, directions, and guiding principles for the study of the resource-based economy.  Figure 1 shows the basic connotation of sustainable development.

Sustainable development is a comprehensive concept involving economy, society, culture, technology, and natural environment. It refers to the development that not only meets the needs of contemporary people but also does not endanger the ability of future generations to meet their own needs. The coordinated operation of the regional system will be affected by multiple factors, which can be divided into two categories: beneficial factors and limiting factors, collectively referred to as nuclear factors and factors. The relationship between these two factors is different, and the development situation is also different. When the leading factors play a dominant role, the development process is reflected in the competition of human activities for the leading factors, and a large number of people have invested in obtaining sufficient material and environment. Due to the stimulation of competition, this stage of development has grown rapidly; the leading factor is gradually consumed, and the emergence of a series of limiting factors hinders the regional development. This generally refers to the economic structure and a form of coordinated development of resources and environment. In order to realize the coordinated development of environmental resources and economy, it is necessary to understand the value measurement of environmental resources. Only by clarifying the value of environmental resources can we better protect environmental resources and realize coordinated development in practice. According to the adaptive spatial division theory, the value of environmental resources lies in its usefulness, scarcity, and conditions of development and utilization.

### 3.2. Research and General Mechanism Analysis of Economic Development in Transition

**Figure 2:**
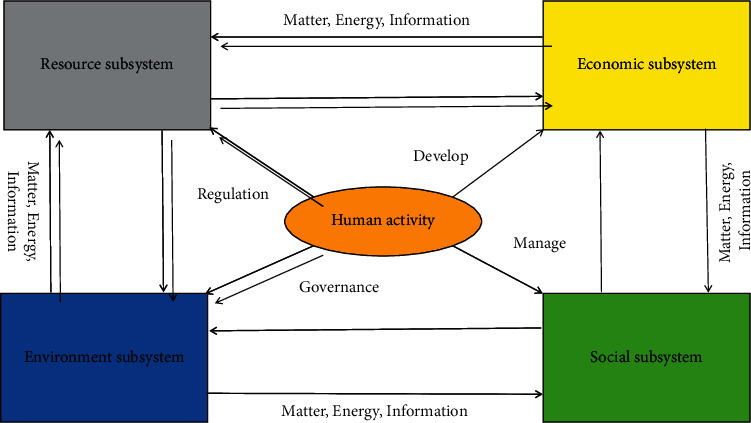
Resource-based economy structure diagram.

The transformation of the resource-based economy is a long-term and complex systematic project. It takes the industrial transformation of the resource-based economy as the starting point and leads and thus triggers social, economic, environmental, and other multifaceted and multilevel changes. Therefore, the transformation of the resource-based economy should be based on the transformation of the industrial structure, change the mode of economic development, and then carry out a comprehensive transformation of the system. In terms of structure, the basic structure of the regional resource environment economic system should be a complex system composed of a resource subsystem, environment subsystem, and economic subsystem. The coupling of the regional resource environment economy system is formed by three levels of system coupling development. First, the internal coupling and coordinated development of a single subsystem. Second, the coupling and coordinated development between the two subsystems. Third, the coupling and coordinated development among the three subsystems.  Figure 2 shows the structure of the resource-based economy.

As shown in Figure 2, a resource-based economy consists of a resource subsystem, an economic subsystem, an environmental subsystem, a social subsystem, and several other factors. Each subsystem is interconnected, influenced, and restricted by human activities, resulting in a crisscross network structure. Furthermore, people are the main body and core of a resource-based economy. People are at the heart of any economic transformation or theory of sustainable development and they are the most important factor in achieving it. As a result, people control the production and distribution of all resources in the network structure depicted in Figure 2. The multiobjective evolutionary algorithm is simple, effective, and has a high degree of generality, making it appealing for solving multiobjective optimization problems. Multiobjective evolutionary algorithms, such as NSGA-II, MOEA/D, MOPSO, and others, are widely used in many fields, including data mining, cloud computing resource management and task scheduling, engineering project scheduling optimization, and Earth observation satellite task planning. This is an evaluation index that is used to determine whether Pareto's optimal solution is reasonable and reliable under self-use space division. Therefore, the objective function is generally introduced to achieve the requirement constraint of space division:

(2) $$\begin{array}{c} \min\left[\sum_{i=1}^{N_{G}}F_{i}\left(P_{Gi}\right),\sum_{i=1}^{N_{G}}E_{i}\left(P_{Gi}\right)\right]. \end{array}$$

**Figure 3:**
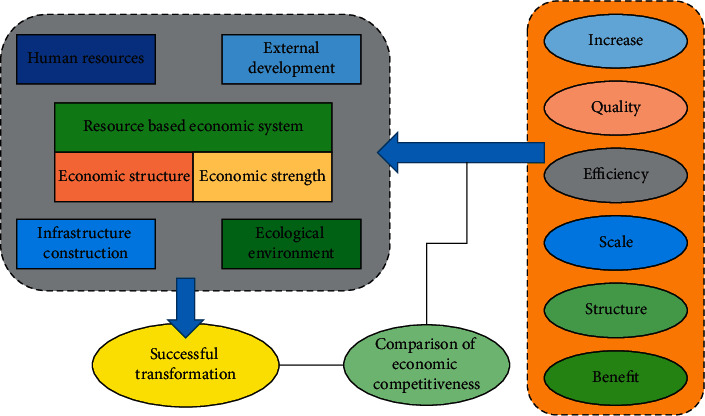
Schematic diagram of the general mechanism of resource-based economic transformation.

Among them: *F*_*i*_(*P*_*Gi*_) is the resource consumption function, *E*_*i*_(*P*_*Gi*_) is the pollution amount function *i*=1,2,…, *N*_*G*_, *N*_*G*_ are the total number of all resources in the space. The Pareto optimal solution of a continuous multiobjective optimization problem is a piecewise continuous dimensional manifold in the objective space. In the process of evolving *m* − 1, if the population lacks diversity, the algorithm may ignore some key search areas and weaken its search ability, making the convergence ability of the algorithm weak or even unable to converge to the real *PF*. Therefore, the general mechanism of resource-based economic transformation can be proposed as shown in Figure 3.

The resource-based economy needs to evolve harmoniously under the action of internal and external forces in order to achieve a successful transformation. Figure 3 lists several key elements of internal and external forces of the resource-based economy. First of all, we should know that scientific and technological strength and innovation are extremely helpful to the promotion of the economy. Different from the mechanism by which capital and labor promote economic growth, a technological progress has an accelerating effect, a delaying effect, a magnifying effect, or a leading effect on economic growth. The infrastructure affects the industrial scale and industrial structure of the economy by affecting the rate of return of economic industries and shaping the ability of the economy to attract talents, technology, capital, resources, and other elements. The construction and improvement of the infrastructure can provide transportation, communication, energy, and power facilities for the optimization of economic and industrial structure and economic development.

### 3.3. Dynamic Model Design for Coordinated Development

Because collecting transition economy samples is difficult, an algorithm model that can expand data processing in the same period must be introduced. As a result, this paper proposes the panel data model for designing and processing the algorithm portion of the model in order to meet the model's design requirements. The panel data model can effectively weaken the mutual influence and differences between various economies, as well as be described in very specific terms in dynamic changes. The model's design accuracy can be improved when multicollinearity weakens. As a result, the following is the fundamental model proposed in this paper:

(3) $$\begin{array}{c} {\text{TRA}}_{it}=c+\alpha_{2}{\text{MAR}}_{i,t}+\alpha_{4}{\text{URB}}_{it}+\alpha_{5}{\text{EDU}}_{i,t}+\alpha_{6}{\text{INF}}_{i,t}+\varepsilon_{it}, \end{array}$$

where *i*, *t* represents the *i* region and *t* year, respectively, and *ε* represents the random interference term. The meaning of each variable and coefficient standard are as follows. *TRA* in the model represents the transformation degree of the resource-based economy. In this paper, the vector angle is used to calculate the industrial structure transformation coefficient, which makes the model quite operable. It not only takes into account the change degree of the same industrial proportion in different years but also reflects the average change degree of the ratio of different industries, which is difficult to achieve by the general coefficient standard. Its specific expression is as follows:

(4) $$\begin{array}{c} \theta =\arccos\left[\frac{\sum_{i=1}^{n}s_{i}\left(t_{1}\right)s_{i}\left(t_{2}\right)}{\sqrt{\sum_{i=1}^{n}s_{i}{\left(t_{1}\right)}^{2}\sum_{i=1}^{n}s_{i}{\left(t_{2}\right)}^{2}}}\right]. \end{array}$$

In the formula, *n* is the number of industrial sectors, *s*_*i*_(*t*_1_) refers to the share of the added value of *i* industrial sector in *t* years in the GDP of *t* years, and *θ* is the angle between *s*(*t*) and *s*(*t* − 1) vectors, which generally becomes the transformation coefficient of the industrial structure. Generally, TEC is expressed as the level of technological progress, and the measurement formula is as follows:

(5) $$\begin{array}{c} \text{TEP}=G-\alpha k-\beta l. \end{array}$$

In the formula (5), *G* represents the average annual growth rate of GDP, *k* · *l* represents the average annual growth rate of capital and labor input, respectively, and *α* · *β* is the elasticity coefficient of capital and labor, respectively. Generally, in economics, the two values are 0.56 and 0.44. If you need to calculate the value of *k*, you need to calculate the capital stock *K*. Its basic formula is as follows:

(6) $$\begin{array}{c} K_{t}=K_{t-1}\left(1-\delta_{t}\right)+I_{t}, \end{array}$$

where *K*_*t*_ · *K*_*t*−1_ represents the capital stock in the *t* year and the *t* − 1 year, *I*_*t*_ represents the material capital investment in the *t* year, and *δ*_*t*_ represents the depreciation rate in the *t* year. Among the many economic factors, the two main factors, labor and capital, should be emphasized. This paper adopts the transformation of the Douglas function:

(7) $$\begin{array}{c} Y=A_{t}K^{\alpha }L^{\beta }. \end{array}$$

By deriving and simplifying both sides of the equation at the same time, we obtain the following:

(8) $$\begin{array}{c} Y=a+\alpha K+\beta L. \end{array}$$

The specific meaning of formula (8)d*Y*/d*t*/*Y*=*Y*, d*A*_*t*_/d*t*/*At*=*a*, d*k*/d*t*/*K*=*K*, d*L*/d*t*=*L* is that the growth rate of economic benefits is determined by technological progress *a*, labor input *L*, and capital *K*. After observation, it can be found that the economic benefits of transformation are proportional to technological progress. After knowing formula (8), the contribution rate can be calculated as follows:

(9) $$\begin{array}{c} E_{A}=\frac{a}{y}\times 100\%, \end{array}$$

where *E*_*A*_ represents the ratio between the annual technological innovation speed and the economic benefit growth speed in transition. The logistic curve is also checked for the transformation of the resource-based economy. Therefore, if *X*(*t*) represents the transformation process of the resource-based economy, the development speed of the model is d*X*/d*t*. Due to the restriction of resource set and environmental capacity, the resource-based economy generally shows a nonexponential growth, which is generally in the form of the following curve:

(10) $$\begin{array}{c} \frac{\text{d}X}{\text{d}t}=\pi X\left(1-\frac{X}{X_{m}}\right). \end{array}$$

Among them, *π* is the growth rate required for development and *X*_*m*_ is the saturated capacity of the development factor. Generally speaking, if the threshold value of the capacity is found, the entire economic system will collapse directly. Therefore, *X*_*m*_ is a key element of transformation and represents reasonable development. The importance of, so continue to discuss *X*_*m*_, which can be obtained after scoring it as follows:

(11) $$\begin{array}{c} X=\frac{X_{m}}{1+Ce^{-m}}, \end{array}$$

where *C* is a constant *C*=*X*_*m*_ − *X*_0_/*X*_0_ > 0 and when *t*⟶*∞*, *X* converges to the threshold *X*_*m*_. When the derivative is 0, we know that,

(12) $$\begin{array}{c} X_{1},X_{2}=X_{m},\\ X_{3}=\frac{X_{m}}{2}. \end{array}$$

It can be found that *X*_1_, *X*_2_ is an extreme situation. When *X*=0, it indicates that the factor base of resource economic development is 0 and *X*=*X*_*m*_ indicates that the development has reached the threshold of the economic system. Through the above algorithm design and improvement, the dynamic change trend of main parameters under the transformed economic model can be obtained, which will be helpful for analysis and decision making.

## 4. Result Analysis and Discussion

**Figure 4:**
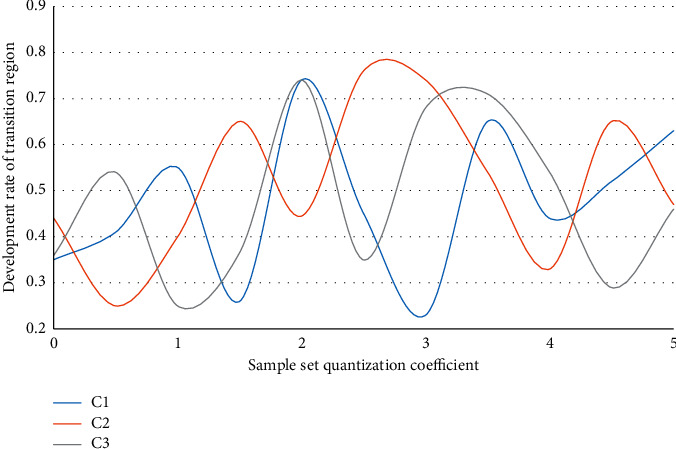
Analysis of the development speed of transitional regions.

**Figure 5:**
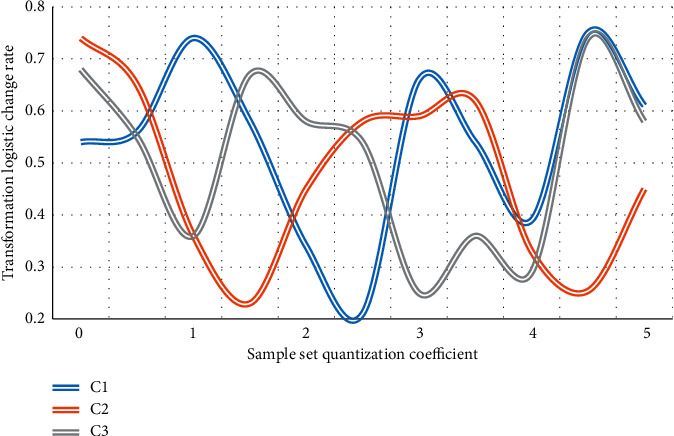
Analysis of the transformation rate of the logistic change.

**Figure 6:**
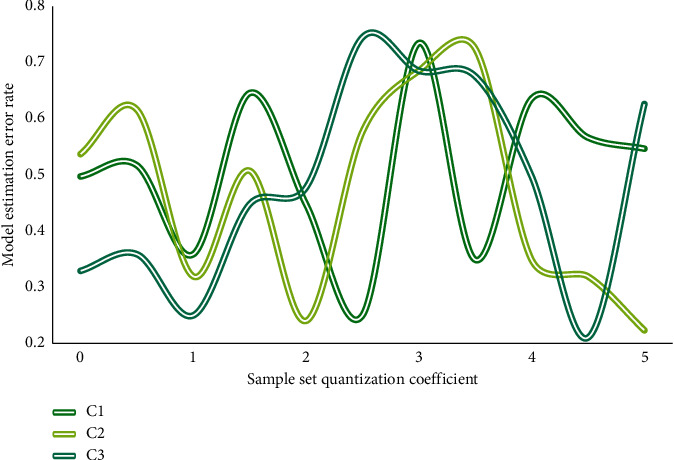
Analysis diagram of the model estimation error rate.

The foundation for the coordinated development of resource-based and resource-environmental economies is the establishment of a scientific, feasible, and practical model system. The coordinated development model of resources, environment, and economy proposed in this paper will examine and address a number of key parameters, including the transition region's development speed, the transition logistics' change rate, the model estimation error rate, the benefit rate of coordinated development, and coordinated development efficiency. Let C1, C2, and C3 be the analysis and comparison data charts of three sample sets of the resource-based economy transformation resource environment economic system in terms of transformation region development speed, transformation logistic change rate, and model estimation error rate as shown in Figures 4–6.

**Figure 7:**
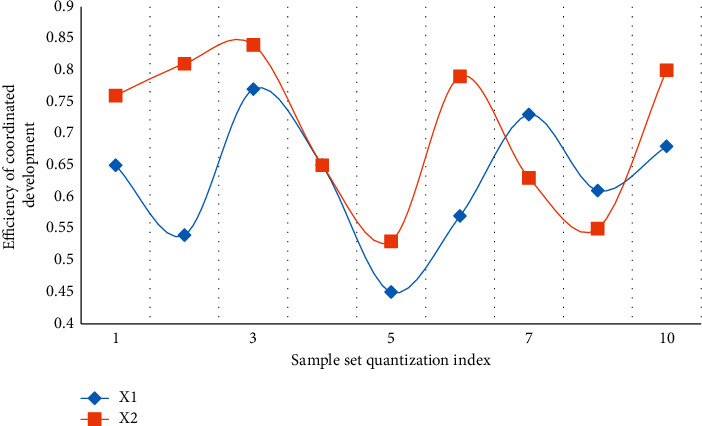
Analysis of the benefit rate of coordinated development.

**Figure 8:**
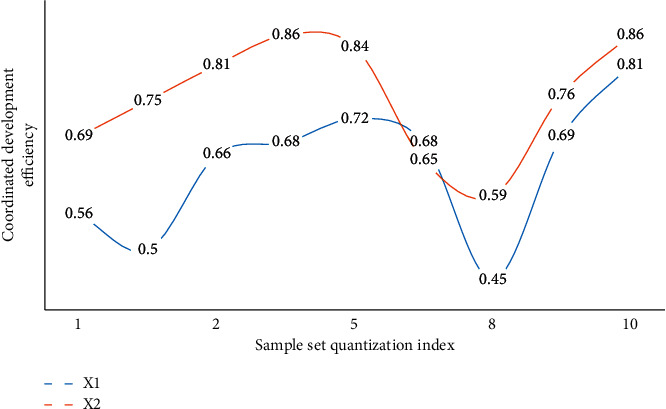
Analysis of coordinated development efficiency.

The abovementioned three indicators are in a stage of greater volatility between 1 and 3 in the above graph due to interference items in the transformation. Of course, this is also foreseeable, and the economic transformation may necessitate a variety of policy adjustments. Market fluctuations, for example, can stymie the transformation process. As a result, while the transformation is risky in practice, the overall trend in the transformation area is upward, implying that despite the presence of interference items, the economic situation is improving. The system will benefit as a whole if it begins to transit from a resource-based economy to a resource-environmental economy with coordinated development. In terms of the logistic change rate, it is discovered that three different sample sets in the entire change process, especially in the 0-1 and 4-5 stages, have the same trend change tendency, proving that the model designed in this paper is universal, and the control of change rate reaches 87.4 percent, greatly improving the model's capture and mastery of unfavorable factors in the transition process. The model's error is analyzed intuitively in the experiment. In the three sample sets, it can be found that the overall error analysis state of sample set C2 is good. It is found that due to the existence of evaluation models for different risks in sample set C2, this also makes the model designed in this paper more accurate after comparing the other two sample sets and having better control over the risk, with a 77.3% improvement in error analysis. Assuming that *X*1 and *X*2 are two different sample sets of coordinated development benefit rate and coordinated development efficiency, Figures 7 and 8 are the analysis diagrams of the two.

From Figures 7 and 8, it can be concluded that the benefit rate of coordinated development is basically kept at a high level, which reflects that the model designed in this paper has a very good promoting effect, and it promotes its rapid growth in the coordinated development of resources, environment and economy in the transformation of the resource-based economy. Although the benefits fluctuate in the 3–5 stages, they quickly return to the high-speed growth level, and the average growth rate of the transformation benefits generally reaches 56.8%. The efficiency of coordinated development is an important reference index. For the region after adaptive spatial division, under the constraints of the model, it basically promotes the development efficiency of the economic system after coordinated transformation. It is worth noting that the stability is basically maintained on the entire quantization axis, which is generally difficult to achieve in practice, but because the panel data model is embedded in the model designed in this paper, the stability of the model is greatly enhanced.

## 5. Conclusions

This paper conducts a systematic, selective, and focused exploratory research and analysis on the sustainable development of a resource-based economy, based on systematically summarizing related research results at home and abroad, with the goal of constructing a theoretical research framework and platform for the coordinated development of resources, environment, and economy in resource-based economy transformation. The goal of resource-based economy industrial development model innovation is to achieve sustainable development, long-term competitiveness, a reasonable economic structure, a healthy ecological environment, and social harmony and stability. We must follow the principles of sustainable development, intensification, environmental protection, and efficiency to achieve this. To achieve a coordinated transformation from disorder to order, the resource-based economy requires a combination of internal and external factors. Three levels of analysis are used to examine the resource-environment-economic subsystem. To begin with, the resource-environment-economy subsystem's basic status is examined from four perspectives: natural environment, resources, economy, and environmental conditions. In terms of adaptive space division, the collaborative model is designed based on important theoretical algorithms such as the panel data model. The error analysis is improved by 77.3% and the average growth rate of transformation benefits is 56.8%.

## Data Availability

The data used to support the findings of this study are available from the corresponding author upon request.
